# Pro-Inflammatory Adipokine and Cytokine Profiles in the Saliva of Obese Patients with Non-Alcoholic Fatty Liver Disease (NAFLD)—A Pilot Study

**DOI:** 10.3390/ijms24032891

**Published:** 2023-02-02

**Authors:** Beata Zyśk, Lucyna Ostrowska, Joanna Smarkusz-Zarzecka, Katarzyna Witczak-Sawczuk, Agnieszka Gornowicz, Anna Bielawska

**Affiliations:** 1 Department of Dietetics and Clinical Nutrition, Medical University of Bialystok, Mieszka I Street 4B, 15-054 Bialystok, Poland; 2 Department of Biotechnology, Medical University of Bialystok, Jana Kilinskiego Street 1, 15-089 Bialystok, Poland

**Keywords:** non-alcoholic fatty liver disease (NAFLD), NAFLD diagnosis, obesity, saliva, NAFLD biomarkers, pro-inflammatory cytokines, resistin, matrix metalloproteinase 9 (MMP-9), matrix metalloproteinase 2 (MMP-2), interleukin 1 β (IL-1β)

## Abstract

Undiagnosed and untreated non-alcoholic fatty liver disease (NAFLD) can lead to the development of many complications, such as cirrhosis, hepatocellular carcinoma, or cardiovascular diseases. Obese people are at increased risk of developing NAFLD. Due to the current lack of routine diagnostics, it is extremely important to look for new diagnostic methods and markers for this disease. The aim of this study was to assess the concentration of selected pro-inflammatory adipokines and cytokines in the unstimulated saliva of obese people with fatty liver disease in various stages (with or without slight fibrosis) and to analyze them for possible use as early markers of NAFLD diagnosis. The study involved 96 people who were divided into 5 groups based on the criterion of body mass index (BMI) and the degree of fatty liver (liver elastography). There were statistically significant differences between the groups in the concentrations of MMP-9 (matrix metalloproteinase 9), resistin, and IL-1β (interleukin 1β) in saliva. Statistically significant, positive correlations between hepatic steatosis and the concentration of MMP-2 (matrix metalloproteinase 2), resistin, and IL-1β in saliva were also found. Statistically significant positive correlations were also found between the concentration of resistin in saliva and the concentration of ALT (alanine aminotransferase) and GGTP (gamma-glutamyl transpeptidase) in serum. MMP-2, IL-1β, and resistin may be potential markers of NAFLD development, assessed in saliva. However, further research is needed because this is the first study to evaluate the concentrations of the selected pro-inflammatory parameters in the saliva of patients with NAFLD.

## 1. Introduction

Non-alcoholic fatty liver disease (NAFLD) is a complex disease that can be subcategorized into two conditions: non-alcoholic fatty liver (NAFL) and non-alcoholic steatohepatitis (NASH). NAFL involves simple steatosis in the absence of inflammation or mild inflammation and a low risk of developing cirrhosis. NASH is a more advanced stage of NAFLD, characterized by fatty liver with chronic, progressive hepatitis with or without fibrosis and a risk of progression to cirrhosis and hepatocellular carcinoma (in the absence of appropriate treatment). Currently, the percentage of people with NAFLD in the world is approximately 25%, of which 1.6–6.5% suffer from NASH in the general population. In addition, there is an association between a higher body mass index (BMI) and the development of NAFLD. Namely, this disease affects more than 90% of people with morbid obesity (BMI ≥ 40 kg/m^2^), and 37% of them have NASH. Moreover, there are data indicating the development of NAFLD in 7–10% of children worldwide [1,2,3]. 

NAFLD is diagnosed when ≥5% of hepatocytes are steatotic, assessed based on histological examination of a liver section, which is considered the gold standard of diagnosis. Liver biopsy enables the differentiation of fatty liver and steatohepatitis, as well as the diagnosis of fibrosis and the analysis of its severity. However, it is an invasive and expensive test, which makes it impossible to use in the routine diagnosis of NAFLD. Ultrasonography (USG) is inexpensive and non-invasive but cannot be considered a good diagnostic alternative for the assessment of hepatic steatosis due to its low sensitivity (the diagnosis of NAFLD with USG is possible only when steatosis exceeds 20% of hepatocytes). In addition, the sensitivity of this method is even lower in obese people. Computed tomography (CT) is more sensitive than ultrasound but is not recommended for routine use due to ionizing radiation. Magnetic resonance imaging (MRI) is a safe and more sensitive method than USG (the diagnosis of NAFLD with MRI is possible with 5–10% hepatocyte steatosis). Additionally, the superiority of this method over USG and CT is based on its ability to quantify fat content. However, it generates high costs. Similarly, a new magnetic resonance imaging technique using the measurement of proton density of the fat fraction (MRI-PDFF) allows for the detection of early-stage fatty liver, but it is not widely available [3,4].

A diagnostic tool that may become an alternative to liver biopsy in the future is FibroScan [5,6], which uses the method of vibration-controlled transient elastography (VCTE). This method allows for both the diagnosis of fatty liver and the analysis of its severity by assessing the controlled attenuation parameter (CAP), as well as the quantitative assessment of liver fibrosis. It is completely safe for patients and non-invasive, as well as sensitive and precise. In addition, the XL probe allows for accurate measurements in people with obesity [7]. The diagnosis of liver fibrosis is also possible with magnetic resonance elastography (MRE), which has very high accuracy but also very low availability. Aspartate aminotransferase (AST) and alanine aminotransferase (ALT), assessed based on laboratory tests, can only be used as additional diagnostics but not as basic ones. Due to the chronic inflammation accompanying NAFLD, the assessment of the concentration of pro-inflammatory substances produced in this process can also be used in its diagnosis [3,4].

A promising direction of scientific research is the evaluation of various pro-inflammatory cytokines and adipokines in terms of their use as potential markers of many diseases [3]. Currently, only a few studies have covered the topic of NAFLD. In addition, the results of the research conducted so far on the use of selected pro-inflammatory substances as markers of NAFLD are not sufficient. Serum is a commonly used material for the determination of pro-inflammatory substances. However, increasingly often in laboratory diagnostics, biological materials are used that are collected in a non-invasive way and do not generate high costs [8]. A good alternative for the determination of pro-inflammatory markers in the blood may be saliva because its composition is very similar to blood plasma [9,10].

Based on the review of the available literature, the following pro-inflammatory adipokines were selected for the analyses in this study: resistin, visfatin, and cytokines, namely tumor necrosis factor α (TNF-α), interleukin 6 (IL-6), interleukin 1β (IL-1β), and matrix metalloproteinases MMP-2 and MMP-9. Resistin participates in the development of inflammation and insulin resistance, which play a key role in the pathogenesis of NAFLD [11]. Visfatin induces oxidative stress in cells and apoptosis. In addition, it has a pro-inflammatory effect and affects lipid and glucose metabolism, as well as the development of insulin resistance [12]. Pro-inflammatory cytokines i.e., TNF-α, IL-6 and IL-1β participate in the progression of simple steatosis to NASH by influencing hepatocyte apoptosis and necrosis, neutrophil chemotaxis, stellate cell activation, and Mallory body production [13]. Therefore, the discussed adipokines and cytokines can be considered in terms of their use as early markers of the development of NAFLD. Changes in the expression of matrix metalloproteinases affect the process of fibrogenesis by participating in the degradation of the extracellular matrix. Liver fibrosis is caused by damage to its connective tissue, i.e., an increase in the amount of fibrous extracellular matrix, with MMP-2 and MMP-9 being particularly important in this process [14]. In our study, we assessed whether matrix metalloproteinases can also be markers of NAFLD in the earlier stages of the disease.

The aim of this study was to assess the concentration of selected pro-inflammatory adipokines and cytokines in unstimulated saliva in patients with obesity and fatty livers in various stages (without fibrosis or with slight fibrosis). Another aim of the study was to analyze pro-inflammatory parameters in terms of their possible use as early markers for the diagnosis of NAFLD.

## 2. Results

**Figure 1:**
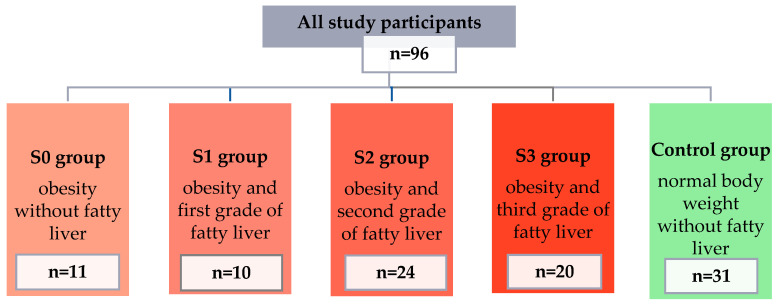
Characteristics of patients qualified for the study, divided into groups.

The concentrations of selected pro-inflammatory adipokines and cytokines were assessed in a group of 96 patients (67 women and 29 men). Participants were divided into 5 groups: 4 study groups (people with obesity and various grades of fatty liver) and a control group (people with normal body weight and no fatty liver). Detailed information on individual groups is presented in Figure 1.

Then, the anthropometric and body composition parameters of all participants were characterized. The groups differed significantly in age (*p* = 0.006). The median of this parameter was 39 years in the S0 group, 44 years in the S1 group, 45.5 years in the S2 group, 45.5 years in the S3 group, and 35 years in the control group. There were also statistically significant differences between the groups for body weight, height, BMI, percentage of total body fat, visceral adipose tissue (VAT) area in the cross-section of the abdomen, and VAT/subcutaneous adipose tissue (SAT) ratio. The results are presented in Table 1.

All participants underwent liver elastography examinations. The median and quartile values for the CAP—controlled attenuation parameter (dB/m) and E—liver stiffness (kPa) are presented in Table 2.

**Figure 2:**
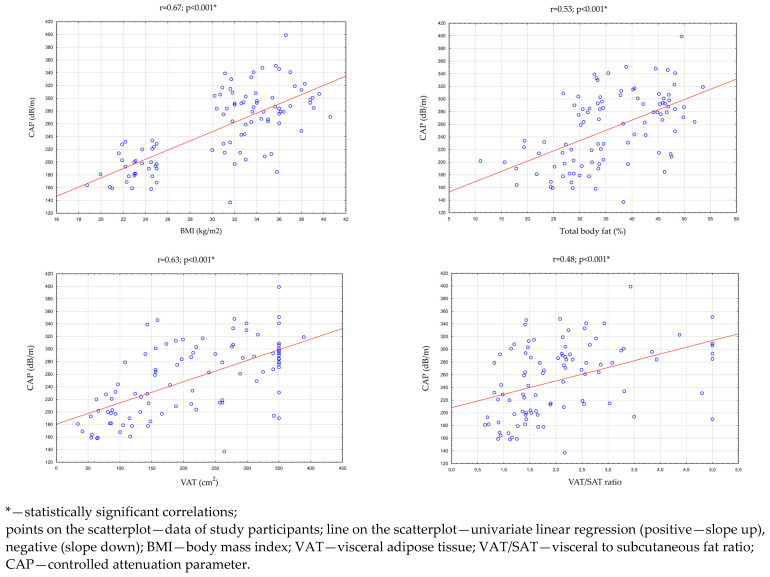
Correlations between the CAP and BMI, total body fat, VAT area in the transverse section of the abdomen, and the VAT/SAT ratio.

Then, an analysis of the correlation between the CAP, BMI index, and parameters evaluating adipose tissue was performed in all study participants (n = 96). There were statistically significant, high positive correlations between the CAP and the BMI index (*p* < 0.001 r = 0.67), the percentage of total body fat (*p* < 0.001; r = 0.53), and the VAT area in cross-section transverse abdomen (*p* < 0.001; r = 0.63). There was also a statistically significant, medium positive correlation between the CAP and the VAT/SAT ratio (*p* < 0.001; r = 0.48). These relationships are presented in Table 3 and Figure 2.

**Figure 3:**
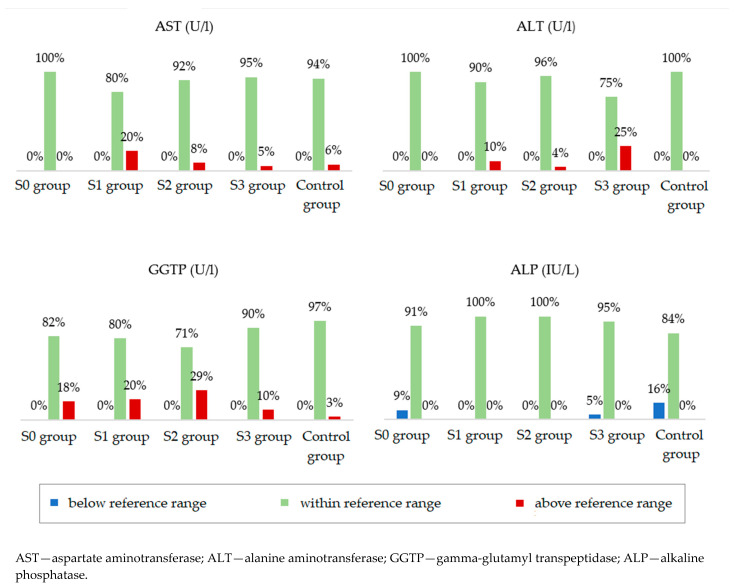
Percentage of people in individual groups who meet the norm for a given laboratory parameter.

Participants from the four study groups and the control group were compared in terms of serum concentrations of liver enzymes (Table 4). Statistically significant differences between the groups were noted for aspartate aminotransferase (AST), alanine aminotransferase (ALT), and gamma-glutamyl transpeptidase (GGTP). The median ALT concentration was the lowest in the group with normal body weight and without hepatic steatosis. However, in the groups with obesity, the median showed higher values with increasing grades of fatty liver. The median concentration of GGTP was lower in the groups without steatosis, both in the study and control groups, in relation to the groups with steatosis. Alkaline phosphatase (ALP) concentrations were not significantly different between the groups. Figure 3 shows the percentages of people whose parameters met the ranges of the accepted standards. Analysis of these data showed that the AST, ALT, GGTP, and ALT concentrations were normal in the majority of patients in all groups. In addition, serum total bilirubin (TBIL) and C-reactive protein (CRP) concentrations were normal in the majority of patients and did not differ depending on the severity of fatty liver disease.

Then, the concentrations of selected pro-inflammatory adipokines and cytokines in saliva were compared among the study groups. The results are presented in Table 5. Statistically significant differences were found for the concentrations of matrix metalloproteinase 9, resistin, and interleukin 1β. However, the concentrations of matrix metalloproteinase 2 and interleukin 6 did not differ significantly between the groups. In addition to the pro-inflammatory parameters presented in Table 5, the concentrations of tumor necrosis factor α and visfatin were also assessed. However, the concentrations of these parameters (in all patients for TNF-α and a large part of the group for visfatin) were below the minimum value recognized by the testing equipment, which prevented analysis.

**Figure 4:**
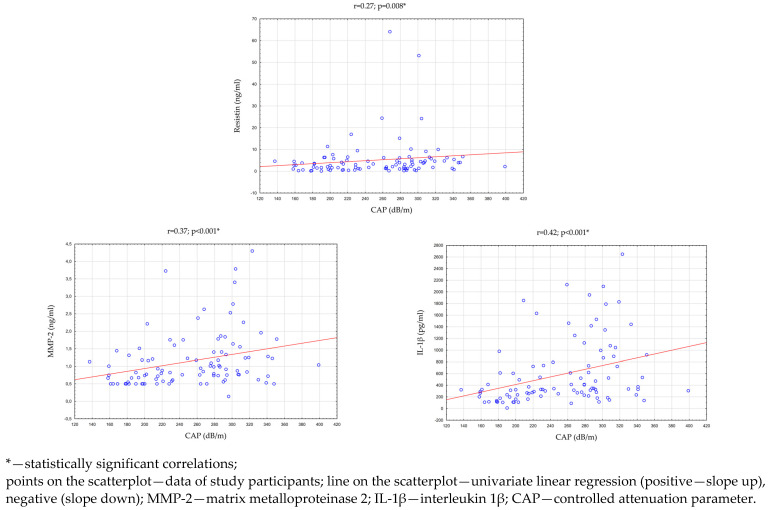
Statistically significant correlations between CAP and the concentration of adipokines and cytokines in saliva.

Then, an analysis of the correlation between the parameters evaluating the clinical condition of the liver and the concentration of adipokines/cytokines in the saliva of all study participants (n = 96) was performed. There were statistically significant, positive correlations between the CAP and the concentration of MMP-2 (*p* < 0.001; r = 0.37), IL-1β (*p* < 0.001; r = 0.42), and resistin (*p* = 0.008; r = 0.27) in saliva. In addition, the concentration of resistin in saliva was statistically significantly correlated with the concentrations of ALT (*p* = 0.032; r = 0.22) and GGTP (*p* = 0.003; r = 0.30) in the serum. The above results are presented in Table 6 and Figure 4.

## 3. Discussion

The high prevalence of NAFLD worldwide and the lack of clinical symptoms before the onset of its late complications (which makes its diagnosis often incidental), as well as the current lack of routine diagnostic tests, mean that the overriding goal should be to look for new diagnostic methods [3]. NAFLD, if not diagnosed and treated early enough, may lead to the development of complications, such as advanced liver fibrosis, cirrhosis, or hepatocellular carcinoma, as well as extrahepatic complications, such as cardiovascular diseases. Studies show that NAFLD may contribute to the development of hypertension, cardiomyopathy, arrhythmias, and ischemic heart disease, and increase the risk of cardiovascular mortality [15].

Due to the fact that obese people are a group predisposed to the development of NAFLD, it would be reasonable to systematically monitor the condition of the liver in terms of the development of steatosis. Currently, the only form of assessment of the clinical condition of the liver is the examination of the serum concentration of liver enzymes, AST, and ALT, as part of routine diagnostics. In our research, the concentrations of AST, ALT, GGTP, ALP, and TBIL in serum were assessed. Although there were statistically significant differences in the concentration of ALT (*p* = 0.002) and GGTP (*p* < 0.001) between the groups, the concentrations of these parameters, as well as other assessed parameters, were within the normal range in the vast majority of people in all groups (regardless of fatty liver severity). Therefore, they are not a good measure of the development of fatty liver disease. The results of studies conducted by other authors [16,17,18,19] also showed that the concentrations of liver enzymes in patients with NAFLD may be normal. Serum C-reactive protein concentration was also assessed in this study, and no significant differences between the groups were found. In addition, the concentrations of this parameter in the majority of study participants (with and without fatty liver) were normal. On the other hand, the authors who assessed serum concentrations of highly sensitive C-reactive protein (hs-CRP) showed that hs-CRP may be a marker of the development and progression of NAFLD [20,21]. The use of more advanced diagnostic methods (USG, MRI, CT) in routine diagnostics is not possible.

NAFLD’s pathogenesis is complex and multifactorial. The development of this disease is influenced by many factors, such as nutritional factors, adipose tissue activity, insulin resistance, changes in the intestinal microbiota, and genetic factors. Chronic inflammation promotes the progression from simple steatosis to steatohepatitis. White adipose tissue (WAT) plays an important role in regulating NASH development by secreting adiponectin, leptin, IL-6, and TNF-α [22]. According to previous studies, SAT is not associated with the development of fatty liver. VAT shows increased metabolic activity and leads to the occurrence of many metabolic disorders, e.g., NAFLD [23]. In addition, the results obtained in this study confirm the relationship between total and visceral adipose tissue and NAFLD development. Significantly higher median VAT area in the transverse section of the abdomen and VAT/SAT ratio were observed in the groups with advanced fatty liver (grades II and III) compared to the group with grade I steatosis and both groups without fatty liver. Moreover, it was observed that as the percentage of total body fat, VAT area in the abdominal cross-section, and VAT/SAT ratio increased, there was an increase in the fatty liver. These correlations were statistically significant (*p* < 0.001).

Leptin may promote the development of inflammation by activating Kupffer cells, which are resident liver macrophages [22]. M1-type macrophages, which are induced by pro-inflammatory mediators, induce the secretion of pro-inflammatory cytokines, e.g., TNF-α, IL-6, and IL-1β. M2-type macrophages have anti-inflammatory activity. Changing the phenotype between M1 and M2 macrophages is an important mechanism in the regulation of inflammatory responses. Inflammatory processes in the liver and damage to hepatocytes cause the activation of hepatic stellate cells (which remain dormant under physiological conditions), which transform them into cells that produce excess collagen and lead to the development of fibrosis [4,24]. In addition to Kupffer cells, during the development of inflammation in NAFLD, liver dendritic cells, neutrophils, T lymphocytes, and NKT cells (natural killer T cells) are involved [22].

Pro-inflammatory adipokines and cytokines show great diagnostic potential in diseases when inflammation is involved in pathogenesis. In studies conducted so far, concentrations of some pro-inflammatory substances in the blood serum of patients with NAFLD have been determined. To our knowledge, our study is the first to assess the profile of selected adipokines and cytokines in the saliva of NAFLD patients. This biological material was chosen because of the promising research results, indicating the possibility of using the determination of some adipokines and cytokines in saliva in the diagnosis of obesity [25,26]. In addition, the results of studies have shown a correlation between the concentration of various substances (including adipokines) in saliva, and their concentrations in blood [27,28,29] confirm the possibility of using saliva as an alternative diagnostic material to blood serum.

Salivary concentrations of MMP-9, MMP-2, resistin, visfatin, IL-6, IL-1β, and TNF-α were assessed in this study. Higher median MMP-2 concentrations were found in groups of people with fatty liver compared to groups of people without fatty livers. However, these results were not statistically significant (*p* = 0.072). However, statistically significant, positive correlations (*p* < 0.001; r = 0.37) were observed between hepatic steatosis and the concentration of MMP-2 in saliva. There are currently no other studies evaluating salivary MMP-2 levels in NAFLD patients. Yilmaz et al. found significant differences in the concentration of MMP-2 in the serum between patients with NAFLD and the group without this disease. However, they concluded that MMP-2 is not a marker that would allow differentiation between simple steatosis and NASH [30]. In studies by Ando et al., which evaluated serum cytokine concentrations in patients with histologically confirmed NASH, MMP-2 concentrations were indicative of the progression of fibrosis. However, they were not characteristic of steatosis or inflammation [31].

The median concentration of MMP-9 in saliva in our study was the highest in the group of people with obesity and without hepatic steatosis and showed lower values in the groups with a higher degree of steatosis. However, its lowest value was recorded in the control group (people without fatty liver and those with normal body weight). Differences between the groups were statistically significant (*p* = 0.016). The authors of other studies assessed only serum MMP-9 concentrations in patients with NAFLD. Goyale et al. showed a lower concentration of MMP-9 in the serum, which was associated with greater liver fibrosis [32]. Livzan et al. showed that the concentration of MMP-9 in blood serum may be a marker of the progression of the initial stages (F1-F2) of liver fibrosis [33]. Wagner et al. conducted a study of obese patients after bariatric surgery. NAFLD (NAS) activity was assessed by liver biopsy. There were no significant differences between the groups in the incidence of liver fibrosis. However, serum MMP-9 concentrations were elevated in NASH patients compared to NAFL and non-NAFLD patients, and borderline patients. Statistically significant correlations between the concentrations of MMP-9 and NAS were found [34]. Yilmaz et al. observed higher concentrations of MMP-9 in the serum of people with NAFLD compared to the control group [30]. Ando et al. did not find a relationship between the serum concentration of MMP-9 and disease activity in a group of patients with NASH [31].

To our knowledge, this study is the first to assess the concentration of IL-1β in saliva in terms of its possible use as a marker of NAFLD. Higher median concentrations of this cytokine were noted in the groups with obesity and fatty liver degrees I, II, and III (including the highest in the group with stage III fatty liver) compared to the groups without fatty liver, both with obesity and with normal body weight. The differences between the groups were statistically significant (*p* = 0.001). In addition, there were statistically significant positive correlations (*p* < 0.001; r = 0.42) between hepatic steatosis and the concentration of IL-1β in saliva. A study by Duan et al. showed that serum IL-1β concentrations were correlated with the development of NAFLD and were associated with NASH, as well as hepatic fibrosis [35]. Vonghia et al. found higher intrahepatic concentrations of this cytokine in patients with obesity and advanced liver fibrosis (F3-F4) [36]. Hadinia et al. compared the concentrations of IL-1β in three groups of patients: people with NAFL, NASH, and people with a normal liver condition. The study showed that IL-1β serum concentrations were significantly higher in patients with NASH compared to those with simple steatosis and the control group [37].

In our study, statistically significant differences (*p* = 0.022) in the median salivary resistin concentrations between the groups were found. Statistically significant positive correlations between hepatic steatosis and resistin concentration in saliva were also found (*p* = 0.008; r = 0.27). In addition, a positive relationship was observed between serum ALT concentration and salivary resistin concentration (*p* = 0.032; r = 0.22). The concentration of resistin in saliva was also correlated with the concentration of GGTP in serum (*p* = 0.003; r = 30). However, no other studies evaluating resistin as a prognostic biomarker of NAFLD have been conducted thus far.

Our research showed no statistically significant differences in the concentration of IL-6 in saliva depending on the presence of steatosis and its severity. There was also no statistically significant correlation between the degree of steatosis and the concentration of IL-6 in saliva. Therefore, perhaps saliva is not a suitable biological material to assess IL-6 concentration regarding NAFLD diagnosis. However, based on studies by other authors, it can be postulated that serum IL-6 may be a good marker of the development and progression of NAFLD. A study by Duan et al. showed that serum concentrations of this cytokine were correlated with the development of NAFLD [35]. In addition, Goyale et al. observed that higher concentrations of IL-6 in blood serum were associated with greater liver fibrosis [32]. Hadinia et al. assessed the concentration of IL-6 in groups of people with NAFL and NASH and people with normal liver conditions. They observed that serum concentrations of this cytokine were significantly higher in patients with NASH compared to those with NAFL and the control group [37].

Our study also assessed the concentration of TNF-α in the saliva of the study participants. However, the levels of this parameter in all patients were below the minimum value recognized by the testing equipment. Therefore, it seems that saliva may not be a suitable diagnostic material for determining TNF-α concentration in patients with NAFLD. However, studies by other authors who assessed TNF-α concentrations in the serum of patients with NAFLD obtained contradictory results. In the study by Hadinia et al., which compared serum TNF-α concentrations in NAFL, NASH, and control subjects, no significant differences were observed [37]. Goyale et al. also found no relationship between the serum levels of this cytokine and NAFLD [32]. However, a review of studies by Duan et al. showed that serum concentrations of this cytokine were associated with NAFLD and were also correlated with the development of NASH, as well as liver fibrosis [35].

In our study, the concentration of visfatin in the saliva of participants in all groups was assessed. Concentrations of visfatin in a significant proportion of participants were below the minimum value, recognizable by the equipment used for testing. Therefore, as was the case for TNF-α, saliva may not be a suitable biological material for the determination of visfatin in NAFLD patients. However, based on studies by other authors, it can be postulated that serum visfatin concentration may prove to be a potential marker of NAFLD development and progression. The results of the study by Qiu et al. showed that reduced serum visfatin concentrations were associated with an increased risk of developing NAFLD [38]. Similarly, Mousavi et al. reported associations between blood visfatin levels and NAFLD [39]. According to the results of studies by Elkabany et al., visfatin may be a promising serum biomarker for monitoring liver diseases in children and adolescents [40]. In contrast, a systematic review and meta-analysis by Ismaiel et al. showed that serum visfatin concentrations are not associated with NAFLD, the presence or severity of fatty liver, or liver fibrosis [12].

## 4. Materials and Methods

The protocols of the observational study were approved by the Bioethics Committee of the Medical University of Bialystok, and consent was obtained for its conduct (no. R-I-002/647/2019, APK.002.468.2020, APK.002.39.2021). Participants received detailed and exhaustive information about the course and purpose of the study, were informed about the possibility of resignation at each stage, and gave their written consent to participate in all components of the study before it began.

### 4.1. Participants 

A total of 120 people (81 women and 39 men) aged 20–55 qualified for the study. The inclusion criterion was the presence of simple obesity (BMI = 30.0–39.9 kg/m^2^), and in the control group—normal body weight (BMI = 18.5–24.9 kg/m^2^). In addition, all participants in the study provided a certificate from a dentist confirming the state of oral health and, more specifically, the absence of periodontal disease and inflammation of the oral cavity. Exclusion criteria were the following diseases and disorders: secondary obesity, alcohol consumption >30 g per week (in men) and >20 g per week (in women), history of viral hepatitis (all types), hepatic cholestasis, history of surgical or pharmacological obesity treatment, eating disorders, diabetes type I and II, exacerbated coronary artery disease, having a pacemaker, hormonal disorders, using hormonal contraception or hormone replacement therapy, pregnancy and lactation, steroid therapy, and antiretroviral therapy. 

The patients qualified for the study had their weight and height measured using a weighing scale (with an accuracy of 0.01 kg) and a height meter (with an accuracy of 0.5 cm) by RADWAG WPT 100/200 OW (Radom, Poland) and BMI was calculated. Liver elastography was also performed to assess the degree of hepatic steatosis and fibrosis.

Due to the resignation of participants at various stages of the project, and consequently the lack of complete studies, 12 people were rejected. In addition, two participants with advanced fibrosis were excluded from the obese group due to the specificity of the study, i.e., searching for early markers of non-alcoholic fatty liver disease. Moreover, from the control group, 10 participants with steatosis and fibrosis of the liver, as well as with metabolic obesity with normal body weight (MONW) syndrome, were eliminated. The control group was composed of participants with a normal clinical condition of the liver and without obesity.

The final group of study participants consisted of 96 people and was divided into 5 groups based on BMI and the criterion of the presence of fatty liver and its severity (S0 group—11 people with obesity and without fatty liver; S1 group—10 people with obesity and grade I steatosis; S2 group—24 people with obesity and grade II steatosis; S3 group—20 people with obesity and grade III steatosis; and the control group—31 people without obesity and steatosis).

### 4.2. Body Composition Analysis

Among the study participants, body composition analysis was performed, and the area of adipose tissue in the abdominal cross-section was determined using the bioelectrical impedance method and the BioScan 920–2 device (Essex, UK). The examination was performed in the morning, and the participants were informed of the necessity of fasting and resignation from intensive physical activity before the examination. Body composition analysis was performed in the supine position. The quantitative analysis of abdominal fat was performed in a standing position, and the electrodes were placed in a horizontal line at the level of the navel.

Based on this study, the following were assessed: total body fat content (%), total body fat mass (kg), total lean body mass (%), total skeletal muscle mass (kg), total body water content (l), as well as extracellular (l) and intracellular (l) water content, and the area of adipose tissue in the transverse section of the abdomen: visceral (VAT-Visceral Adipose Tissue, cm^2^, %) and subcutaneous (SAT-Subcutaneous Adipose Tissue, cm^2^, %). In addition, the visceral to subcutaneous fat ratio (VAT/SAT ratio) was established.

The obtained results were processed using the Maltron BioScan 920 v1.1 software.

### 4.3. Measurement of Liver Stiffness and Steatosis Using Dynamic Elastography 

The measurement of liver stiffness (E) was performed using the Echosens FibroScan 530 compact (Paris, France), which also has the option of evaluating the controlled attenuation parameter (CAP). The study was conducted in the summer period (July–September).

Liver stiffness (correlated with fibrosis) is calculated based on the speed of propagation of the shear wave several centimeters into the liver. The transverse wave is generated at a controlled frequency (50 Hz) of a characteristic and specific parameter for determining the stiffness of the liver. The CAP^®^ measurement (evaluating fatty liver), based on VCTE^®^ technology, is performed simultaneously with the liver stiffness measurement. This parameter is determined by measuring the phenomenon of ultrasonic attenuation and is expressed in dB/m (decibel/meter). It describes the decay of the ultrasound signal, depending on the depth of penetration in the examined liver.

The following ranges were adopted for the CAP: 0–10% hepatocyte steatosis—no fatty liver; 11–33%—1st-grade hepatocyte steatosis; 34–66%—2nd-grade hepatocyte steatosis; and ≥67% hepatocyte steatosis—3rd-grade steatosis. The ranges of the parameters evaluating liver stiffness (E) were as follows: F0 and F1—no liver fibrosis; F2—slight fibrosis; F3 and F4—advanced liver fibrosis.

The test was carried out by trained operators. Participants were advised to perform the test on fasting or at least 6 h after a meal. In addition, during the examination, they were instructed on what position to take (lying position with the right arm raised and placed behind the head). The appropriate amount of gel was then applied. The camera head was placed between the ribs at the level of the thickest layer of the liver parenchyma to avoid measurement near the lower or upper edge of the liver (possibility of overestimating the measurement results in the subcapsular area). Head sizes M and XL were used. For each participant, 10 normal liver stiffness measurements were taken at the same measurement point to ensure the repeatability and accuracy of the measurements.

### 4.4. Evaluation of Laboratory Parameters Related to Liver Function

Laboratory tests were performed during the period of July–September (a similar time range as in the case of elastography to be reliable and credible for further analyses). Ten milliliters of blood was collected after fasting. All biochemical assessments were performed in the same laboratory using standard methods.

Serum concentrations of AST, ALT, GGTP, ALP, TBIL and C-reactive protein were assessed. The reference ranges established by the laboratory where the tests were performed were taken into account for these parameters and were as follows: AST, 5–34 U/l; ALT, 0–55 U/L; GGTP, 9–36 U/l (women) and 12–64 U/l (men); ALP, 40–150 IU/L; TBIL, 0.2–1.2 mg/dL; C-reactive protein, 0–10 mg/L.

### 4.5. Saliva Collection 

Saliva was collected using the standard method. Samples from study participants were collected between 9:00–11:00 a.m. Participants were instructed to maintain a minimum of 2 h between eating and drinking (other than pure water) and collecting saliva. Before saliva collection, the oral cavity was rinsed with deionized water. During the study, participants sat in a comfortable position with their heads slightly tilted downward. Unstimulated saliva was collected for 10 min by the spitting method described by Navazesh [41]. Saliva samples were homogenized and clarified by centrifugation at 1200 RPMI for 15 min at 4 °C. The aliquots of clarified supernatants were stored at −70 °C for the ELISA measurements. 

### 4.6. Analysis of Salivary MMP-9, MMP-2, Resistin, Visfatin, IL-6, IL-1β and TNF-α Concentration 

Highly sensitive assay kits (R&D Systems Inc., Minneapolis, MN, USA) were used to measure the concentrations of proteins in the salivary samples of the control and experimental subjects. The microtiter plates provided in the kits were pre-coated with an antibody specific to the analyzed antigen. The tests were carried out according to the manufacturer’s protocols.

### 4.7. Statistical Methods

Statistical analysis was performed using STATISTICA 13.3 software (StatSoft, Cracow, Poland). The significance level was *p* < 0.05. The values of the median, as well as the lower and upper quartiles, were determined. Comparative analysis of salivary adipokines and cytokines between groups was performed using the Kruskal–Wallis ANOVA and the median test. The assessment of the relationship between the concentration of pro-inflammatory parameters in saliva and hepatic laboratory parameters in serum and the CAP of elastography was performed using Spearman’s rank correlation. Non-parametric methods were used due to the lack of normality of the distribution.

## 5. Conclusions

The results of this study indicate an important direction in the diagnosis of non-alcoholic fatty liver disease, i.e., the use of adipokines and cytokines as biomarkers of this disease. The obtained results showed that potential markers of NAFLD development may include: MMP-2, IL-1β and resistin, determined in saliva. However, to the best of our knowledge, our study is the first to assess the concentration of selected pro-inflammatory parameters in saliva among people with obesity and non-alcoholic fatty liver disease. Therefore, further studies on a larger group of patients are necessary, the aim of which should be both to unambiguously determine whether the given adipokines and cytokines can be used as biomarkers of NAFLD, as well as to confirm whether saliva can be used as an alternative diagnostic material for blood serum. It should be noted that blood serum is a more homogeneous material than saliva, but saliva collection is a non-invasive procedure. In our next study, we plan to focus on pro-inflammatory cytokines and adipokines in serum, and characteristic/comparing on both materials.

## Figures and Tables

**Table 1:** Characteristics of anthropometric parameters and body composition of all groups.

Parameter	S0 Group		S1 Group		S2 Group		S3 Group		Control Group		*p*
	Median	Q1–Q3	Median	Q1–Q3	Median	Q1–Q3	Median	Q1–Q3	Median	Q1–Q3	
Body weight (kg)	95.00	86.00–99.70	100.20	91.50–106.00	97.50	92.00–110.55	103.00	95.00–113.00	64.00	58.00–70.00	<0.001*
Height (cm)	166.50	157.00–177.50	170.50	167.00–174.00	166.50	163.00–177.00	171.75	167.50–182.50	163.00	160.00–172.00	0.070 *
BMI (kg/m^2^)	32.00	31.10–34.70	33.95	32.90–35.00	35.00	32.70–36.70	33.80	31.66–36.80	23.10	22.19–24.60	<0.001*
Total body fat (%)	39.22	33.52–46.21	41.44	33.66–45.82	44.48	33.64–46.47	39.54	33.68–46.93	27.34	22.19–30.39	<0.001*
VAT (cm^2^)	220.00	148.00–261.00	209.50	156.00–315.00	345.50	213.50–350.50	279.00	209.50–350.00	88.00	65.00–132.00	<0.001 *
VAT/SAT	1.90	1.42–2.51	1.76	1.41–2.49	2.19	1.87–3.17	2.40	1.53–3.18	1.36	0.94–1.62	<0.001 *

Q1–Q3—1st–3rd lower and upper quartiles; *p* *—statistically significant differences between the groups (*p* < 0.005); BMI—body mass index; VAT—visceral adipose tissue; VAT/SAT—visceral to subcutaneous fat ratio.

**Table 2:** Characteristics of the parameters of the elastography examination (FibroScan) of all groups.

Parameter	S0 Group		S1 Group		S2 Group		S3 Group		Control Group		*p*
	Median	Q1–Q3	Median	Q1–Q3	Median	Q1–Q3	Median	Q1–Q3	Median	Q1–Q3	
CAP (dB/m)	213.00	197.00–219.00	262.00	249.00–264.00	286.50	279.00–293.00	321.00	308.50–341.00	193.00	178.00–214.00	<0.001 *
E (kPa)	4.10	2.80–6.20	4.95	4.00–6.00	5.15	3.85–6.00	5.75	5.10–6.40	3.80	3.30–5.00	<0.001 *

Q1–Q3—1st–3rd lower and upper quartiles; *p* *—statistically significant differences between the groups (*p* < 0.005); CAP—controlled attenuation parameter; E—liver stiffness.

**Table 3:** Correlations between the CAP and the parameters evaluating adipose tissue.

Parameter	BMI (kg/m^2^)	Total Body Fat (%)	VAT (cm^2^)	VAT/SAT
CAP (dB/m)	r = 0.67	r = 0.53	r = 0.63	r = 0.48
	*p* < 0.001 *	*p* < 0.001 *	*p* < 0.001 *	*p* < 0.001 *

*p* *—statistically significant correlations (*p* < 0.005); BMI—body mass index; VAT—visceral adipose tissue; VAT/SAT—visceral to subcutaneous fat ratio; CAP—controlled attenuation parameter.

**Table 4:** Characteristics of the hepatic laboratory parameters of all groups.

Parameter	S0 Group		S1 Group		S2 Group		S3 Group		Control Group		*p*
	Median	Q1–Q3	Median	Q1–Q3	Median	Q1–Q3	Median	Q1–Q3	Median	Q1–Q3	
AST (U/L)	20.00	18.00–26.00	21.50	16.00–27.00	18.50	15.00–26.00	21.50	17.00–27.00	17.00	16.00–19.00	0.018 *
ALT (U/L)	23.00	15.00–26.00	24.00	17.00–29.00	24.50	18.00–31.00	30.50	21.00–53.00	14.00	12.00–19.00	0.002 *
GGTP (U/L)	18.00	14.00–28.00	31.00	20.00–36.00	24.50	18.50–44.50	32.00	21.00–41.00	15.00	11.00–17.00	<0.001 *
ALP (U/L)	52.00	48.00–59.00	68.50	61.00–80.00	58.00	51.50–67.00	60.50	43.50–69.00	52.00	44.00–63.00	0.053

Q1–Q3—1st–3rd lower and upper quartiles; *p* *—statistically significant differences between the groups (*p* < 0.005); AST—aspartate aminotransferase; ALT—alanine aminotransferase; GGTP—gamma-glutamyl transpeptidase; ALP—alkaline phosphatase.

**Table 5:** Characteristics of the concentration of selected adipokines and pro-inflammatory cytokines in the unstimulated saliva of the study groups and the control group.

Parameter	S0 Group		S1 Group		S2 Group		S3 Group		Control Group		*p*
	Median	Q1–Q3	Median	Q1-Q3	Median	Q1–Q3	Median	Q1–Q3	Median	Q1–Q3	
MMP-9 (ng/mL)	573.00	445.70–690.60	522.90	431.80–1335.80	391.60	239.45–714.05	369.00	315.50–614.45	265.80	124.50–475.00	0.016 *
MMP-2 (ng/mL)	0.80	0.64–1.13	1.06	0.76–1.76	1.02	0.75–1.52	1.23	0.77–1.87	0.67	0.50–1.18	0.072
Resistin (ng/mL)	3.35	1.71–5.04	2.69	1.36–6.25	2.65	1.21–4.98	4.69	3.87–6.37	1.71	0.56–3.83	0.022 *
IL-6 (pg/mL)	7.15	3.13–12.74	8.24	6.78–22.24	11.48	5.93–15.06	9.13	4.34–15.92	8.25	3.13–33.72	0.600
IL-1β (pg/mL)	275.48	213.34–373.12	512.27	316.00–1253.01	411.30	281.31–932.24	628.59	319.00–1212.30	266.43	135.35–413.18	0.001 *

Q1–Q3—1st–3rd lower and upper quartiles; *p* *—statistically significant differences between the groups (*p* < 0.005); MMP-9—matrix metalloproteinase 9; IL-6—interleukin 6; MMP-2—matrix metalloproteinase 2; IL-1β—interleukin 1β.

**Table 6:** Correlations between parameters evaluating liver condition and adipokines and cytokines in unstimulated saliva of all persons qualified for the study.

Parameter	MMP-9 (ng/mL)	MMP-2 (ng/mL)	Resistin (ng/mL)	IL-6 (pg/mL)	IL-1β (pg/mL)
CAP (dB/m)	r = 0.11	r = 0.37	r = 0.27	r = 0.10	r = 0.42
	*p* = 0.266	*p* < 0.001 *	*p* = 0.008 *	*p* = 0.333	*p* < 0.001 *
AST (U/L)	r = 0.01	r = 0.11	r = 0.01	r = −0.10	r = −0.04
	*p* = 0.920	*p* = 0.285	*p* = 0.889	*p* = 0.335	*p* = 0.733
ALT (U/L)	r = 0.11	r = 0.20	r = 0.22	r = −0.06	r = 0.12
	*p* = 0.302	*p* = 0.053	*p* = 0.032 *	*p* = 0.547	*p* = 0.228
GGTP (U/L)	r = 0.10	r = 0.10	r = 0.30	r = 0.05	r = 0.15
	*p* = 0.35	*p* = 0.311	*p* = 0.003 *	*p* = 0.645	*p* = 0.146
ALP (IU/L)	r = −0.14	r = 0.14	r = −0.07	r = 0.08	r = 0.08
	*p* = 0.169	*p* = 0.171	*p* = 0.474	*p* = 0.441	*p* = 0.425

*p* *—statistically significant correlations (*p* < 0.005); MMP-9—matrix metalloproteinase 9; IL-6—interleukin 6; MMP-2—matrix metalloproteinase 2; IL-1β—interleukin 1β; CAP—controlled attenuation parameter AST—aspartate aminotransferase; ALT—alanine aminotransferase; GGTP—gamma-glutamyl transpeptidase; ALP—alkaline phosphatase.

## Data Availability

Data available on request due to restrictions, e.g., privacy or ethical.
